# Supplementary material for: Domains of the autism phenotype, cognitive control, and rumination as transdiagnostic predictors of DSM-5 suicide risk
Source: PLoS One. 2021 Jan 22;16(1):e0245562. doi: 10.1371/journal.pone.0245562 (PMC7822649; doi:10.1371/journal.pone.0245562)
Supplement: S1 Appendix — Items for each of the constructs used in the study. (DOCX) [file pone.0245562.s001.docx]

**S1 Appendix. Scale items and ratings.** Items for each of the constructs used in the study.

| Construct | Item | Rating | Original scale |
| --- | --- | --- | --- |
| **Social Communication Difficulties** | I find it easy to read between the lines when someone is talking to me | 4-point Likert-type; 1= “definitely agree”, 4 = “definitely disagree”; Score range = 5–25^a^ | AQ-10 |
|  | I know how to tell if someone listening to me is getting bored |  |  |
|  | When I’m reading a story I find it difficult to work out the characters’ intentions (rev) |  |  |
|  | I find it easy to work out what someone is thinking or feeling just by looking at their face |  |  |
|  | I find it difficult to work out people’s intentions (rev) |  |  |
| **Insistence on Sameness** | Do you prefer to do things in a particular order? | 5-point Likert-type; 1 = “not at all/never”, 5 = “very much/always”; 15-item score range = 15–75 | ARI |
|  | Do you prefer to do things in a certain way? |  |  |
|  | Do you arrange objects or perform certain behaviors until they are "just right?" |  |  |
|  | Do you like to eat your meals in a certain order or certain way? |  |  |
|  | Do you have persistent habits? |  |  |
|  | Do you prefer to keep the same schedule or routine every day? |  |  |
|  | Do you prefer to have certain belongings "in their place?" |  |  |
|  | Do you prefer to finish one task before moving on to the next? |  |  |
|  | Do you prepare for bedtime by engaging in a routine? |  |  |
|  | Do things have to be "in their place" before you can get anything else done? |  |  |
|  | Do you feel you have to complete a task once you have started it? |  |  |
|  | Do you feel bothered if something disrupts your daily schedule or routine? |  |  |
|  | Do you order the same meals when you go to a particular restaurant? |  |  |
|  | Once something is done a certain way do you feel like it has to be done that way every time? |  |  |
|  | Do you insist that certain activities need to take place at a certain time? |  |  |
| **Attentional Control** | It’s often hard for me to alternate between two different tasks (rev) | 7-point Likert-type; 1 = “extremely untrue of you”, 7 = “extremely true of you”; Score range = 5-35 | ATQ |
|  | When I am trying to focus my attention, I am easily distracted (rev) |  |  |
|  | When interrupted or distracted, I usually can easily shift my attention back to whatever I was doing before |  |  |
|  | It is very hard for me to focus my attention when I am distressed (rev) |  |  |
|  | When I am happy and excited about an upcoming event, I have a hard time focusing my attention on tasks that require concentration (rev) |  |  |
| **Inhibitory Control** | Even when I feel energized, I can usually sit still without much trouble if it’s necessary | 7-point Likert-type; 1 = “extremely untrue of you”, 7 = “extremely true of you”; Score range = 7-49 | ATQ |
|  | It is easy for me to hold back my laughter in a situation when laughter wouldn't be appropriate |  |  |
|  | I can easily resist talking out of turn, even when I’m excited and want to express an idea |  |  |
|  | I usually have trouble resisting my cravings for food drink, etc. (rev) |  |  |
|  | When I'm excited about something, it's usually hard for me to resist jumping right into it before I've considered the possible consequences (rev) |  |  |
|  | When I see an attractive item in a store, it’s usually very hard for me to resist buying it (rev) |  |  |
|  | It is easy for me to inhibit fun behavior that would be inappropriate |  |  |
| **Rumination** | Many situations make me worry. | 7-point Likert-type; 1 = “extremely untrue”, 7 = “extremely true”; Responses 1 and 2 are both scored 1; Score range = 3–18 | PSWQ-3 |
|  | Once I start worrying, I cannot stop. |  |  |
|  | I worry all the time. |  |  |
| **Depression** | Little interest or pleasure in doing things? (last 2 weeks) | 5-point Likert-type; 0 = “none or not at all”, 4 = “severe of nearly every day”; Score range = 0–8 | DSM-5 CC |
|  | Feeling down, depressed, or hopeless? (last 2 weeks) |  |  |
| **Suicide Risk** | Thoughts of actually hurting yourself? (last 2 weeks) | Score range 0–4 | DSM-5 CC |

AQ-10: Autism Spectrum Quotient, 10-item (Allison, Auyeung, & Baron-Cohen, 2012); ARI: Adult Routines Inventory (D. W. Evans, Uljarević, Lusk, Loth, & Frazier, 2017); ATQ: Adult Temperament Questionnaire (D. E. Evans & Rothbart, 2007); PSWQ-3: Penn State Worry Questionnaire, ultra-brief version (Berle et al., 2011); DSM-5 CC: DSM-5 Level 1 Cross-Cutting Symptom Measure (Narrow et al., 2013; Narrow & Kuhl, 2011).

^a^The full score range (i.e., 1–4) was retained to maximize score variance.
