# Supplementary material for: Domains of the autism phenotype, cognitive control, and rumination as transdiagnostic predictors of DSM-5 suicide risk
Source: PLoS One. 2021 Jan 22;16(1):e0245562. doi: 10.1371/journal.pone.0245562 (PMC7822649; doi:10.1371/journal.pone.0245562)
Supplement: S1 Table — Distribution of participants between US States and the District of Columbia. (DOCX) [file pone.0245562.s002.docx]

**S1 Table. Participant distribution.** Distribution of participants between US States and the District of Columbia.

| State | *n* | % |
| --- | --- | --- |
| AL | 22 | 1.2% |
| AK | 3 | 0.2% |
| AZ | 32 | 1.7% |
| AR | 19 | 1.0% |
| CA | 203 | 11% |
| CO | 27 | 1.5% |
| CT | 20 | 1.1% |
| DE | 8 | 0.4% |
| FL | 113 | 6.1% |
| GA | 55 | 3.0% |
| HI | 12 | 0.6% |
| ID | 12 | 0.6% |
| IL | 72 | 3.9% |
| IN | 35 | 1.9% |
| IA | 25 | 1.4% |
| KS | 17 | 0.9% |
| KY | 42 | 2.3% |
| LA | 20 | 1.1% |
| ME | 10 | 0.5% |
| MD | 31 | 1.7% |
| MA | 52 | 2.8% |
| MI | 56 | 3.0% |
| MN | 21 | 1.1% |
| MS | 15 | 0.8% |
| MO | 36 | 1.9% |
| MT | 6 | 0.3% |
| NE | 11 | 0.6% |
| NV | 28 | 1.5% |
| NH | 5 | 0.3% |
| NJ | 33 | 1.8% |
| NM | 6 | 0.3% |
| NY | 131 | 7.1% |
| NC | 59 | 3.2% |
| ND | 4 | 0.2% |
| OH | 91 | 4.9% |
| OK | 27 | 1.5% |
| OR | 25 | 1.4% |
| PA | 82 | 4.4% |
| RI | 4 | 0.2% |
| SC | 20 | 1.1% |
| SD | 5 | 0.3% |
| TN | 45 | 2.4% |
| TX | 108 | 5.8% |
| UT | 20 | 1.1% |
| VT | 2 | 0.1% |
| VA | 68 | 3.7% |
| WA | 33 | 1.8% |
| WV | 7 | 0.4% |
| WI | 29 | 1.6% |
| WY | 5 | 0.3% |
| DC | 1 | 0.1% |
| Not reported | 38 | 2.1% |
