# Supplementary material for: Domains of the autism phenotype, cognitive control, and rumination as transdiagnostic predictors of DSM-5 suicide risk
Source: PLoS One. 2021 Jan 22;16(1):e0245562. doi: 10.1371/journal.pone.0245562 (PMC7822649; doi:10.1371/journal.pone.0245562)
Supplement: Attachment — Submitted filename: Review of Manuscript from PLOS ONE.pdf [file pone.0245562.s003.pdf]

This paper examines the relationship between autistic traits and suicidal ideation. I think the topic of this research is very important, and the results of this research can help us better understand both autism and suicidal thoughts. In addition, the study had a very large sample size (n=1851) with participants from a diverse background. Such large representative data can give us accurate estimates of the population parameters of interest; thus, the authors should definitely find a channel to publish these data. However, there are several major problems with the paper that need to be addressed:

1. The data analyses of the paper are not well done. The authors claim that they did hierarchical regression analyses but based on the description of the analysis procedure, they actually did stepwise regression (see page 20). Stepwise regression is usually used in exploratory studies where researchers try to search for important predictors for the criterion variable. However, this paper is clearly a confirmatory study. I don't see any justification for using the stepwise regression. I recommend the authors just use regular multiple regression analyses.
2. The results section of the paper is very poorly written. The results section merely introduced the tables for the results; it did not describe the patterns of the results at all. In the next revision, please provide a summary of the results of each table and describe the results patterns in words (i.e., do not just present the results in tables).
3. The authors included a lot of tables (e.g., Tables 1, 2 and 3) that are not essential for addressing the research goal. I recommend the authors put these tables in the supplementary materials.

In addition, there are few minor problems/mistakes in the paper:

- On page 6, there is a sentence that says "ASD traits were found to significantly correlate with suicidal behaviour, and the relationship was mediated by burdensomeness and thwarted belonging….. " Does it mean burdensomeness and thwarted belonging are two of the underlying mechanisms explaining the relationship between autism and suicidal thoughts? If yes, then why this is not addressed in the next section of the paper, which is about "mechanisms underspinning suicide risk and the autistic phenotype"?
- There is a typo on page 8 under the "Participants" section. It says "…. condiucted recruitment online using…. " "Condiucted" is a typo.
- On page 15, please provide a citation for Little's MCAR test. Also on page 15, I am confused why the authors found out that the data are not MCAR but still used listwise deletion.
- On page 8, the sentence, "The aims of the present study were to examine (1) the contribution of two key clinical domains—social communication difficulties, insistence on sameness—usually considered core features of ASD, but also present across a range of other disorders, that have been associated with suicidal risk and behavior, thereby deconstructing the impact of specific ASD domains on suicide risk (assessed using DSM-5 suicidal ideation; SI)….", is very confusing to read. Please simplify the sentence.
