# Supplementary material for: Domains of the autism phenotype, cognitive control, and rumination as transdiagnostic predictors of DSM-5 suicide risk
Source: PLoS One. 2021 Jan 22;16(1):e0245562. doi: 10.1371/journal.pone.0245562 (PMC7822649; doi:10.1371/journal.pone.0245562)
Supplement: Attachment — Submitted filename: Response to reviewers.docx [file pone.0245562.s004.docx]

Dear editor,

Thank you for the opportunity to revise and re-submit our manuscript. Please find below a detailed response to all comments. We thank the reviewer and the editor for their suggestion leading to what we believe is an improved manuscript. Of note, we have revised the Results section and analyses as per the suggestions of the Review, and appreciate the suggestions.

1. Please ensure that your manuscript meets PLOS ONE's style requirements, including those for file naming. The PLOS ONE style templates can be found at

https://journals.plos.org/plosone/s/file?id=wjVg/PLOSOne_formatting_sample_main_body.pdf and

https://journals.plos.org/plosone/s/file?id=ba62/PLOSOne_formatting_sample_title_authors_affiliations.pdf

**Response:** We have carefully checked the manuscript against the PLOS ONE style templates and have revised the manuscript accordingly, including the reference list, title page, and table notes.

2.Please provide additional details regarding participant consent. In the ethics statement in the Methods and online submission information, please ensure that you have specified (1) whether consent was informed and (2) what type you obtained (for instance, written or verbal, and if verbal, how it was documented and witnessed). If your study included minors, state whether you obtained consent from parents or guardians. If the need for consent was waived by the ethics committee, please include this information. If you are reporting a retrospective study of medical records or archived samples, please ensure that you have discussed whether all data were fully anonymized before you accessed them and/or whether the IRB or ethics committee waived the requirement for informed consent. If patients provided informed written consent to have data from their medical records used in research, please include this information.

**Response:** The study was online. Participants read an information statement about the study and provided online consent. These additional details have been added to the Method section and the Ethics Statement in editorialmanager, and the Cover Letter. The consent process now reads:

“All participants reviewed an information document and were informed that participation was voluntary prior to agreeing to participate in the study. Online consent was received from all study participants.”

3.We note that you have indicated that data from this study are available upon request. PLOS only allows data to be available upon request if there are legal or ethical restrictions on sharing data publicly. For more information on unacceptable data access restrictions, please see http://journals.plos.org/plosone/s/data-availability#loc-unacceptable-data-access-restrictions.

In your revised cover letter, please address the following prompts:

a) If there are ethical or legal restrictions on sharing a de-identified data set, please explain them in detail (e.g., data contain potentially sensitive information, data are owned by a third-party organization, etc.) and who has imposed them (e.g., an ethics committee). Please also provide contact information for a data access committee, ethics committee, or other institutional body to which data requests may be sent. b) If there are no restrictions, please upload the minimal anonymized data set necessary to replicate your study findings as either Supporting Information files or to a stable, public repository and provide us with the relevant URLs, DOIs, or accession numbers. For a list of acceptable repositories, please see <http://journals.plos.org/plosone/s/data-availability#loc-recommended-repositories>. We will update your Data Availability statement on your behalf to reflect the information you provide.

**Response:** All data are fully available without restrictions. Data are freely downloadable from Figshare, <https://doi.org/10.26181/5e992fc659d7c>. The reference for the dataset is:

“Hedley D, Uljarević M, Cai RY, Bury SM, Stokes MA, Evans DW. Transdiagnostic predictors of dsm-5 suicide risk [raw dataset, Version 2]. Figshare. 2020. https://doi.org/10.26181/5e992fc659d7c”

The statement in editorialmanager has been updated to indicate that data are openly available and that additional queries concerning the data sample can be directed to author DWE.

“The data that support the findings of this study are openly available in “figshare” at https://doi.org/10.26181/5e992fc659d7c [89]. Additional correspondence or queries concerning the data sample should be directed to David W. Evans, PhD, Department of Psychology, Bucknell University, Lewisburg, PA, 17837; e-mail: dwevans@bucknell.edu.”

4.Thank you for stating the following in the Competing Interests section:

[I have read the journal's policy and the authors of this manuscript have the following competing interests: DH is supported by a Suicide Prevention Australia National Suicide Prevention Research fellowship. MU is supported by a Discovery Early Career Researcher Award from the Australian Research Council (DE180100632). DWE declares that he receives funding from Roche Pharmaceutical for consulting on the use of the Childhood Routines Inventory-Revised (CRI-R). ].

Please confirm that this does not alter your adherence to all PLOS ONE policies on sharing data and materials, by including the following statement: "This does not alter our adherence to PLOS ONE policies on sharing data and materials.” (as detailed online in our guide for authors https://protect-au.mimecast.com/s/dHriC4QZgRFApQopfjbrgz?domain=journals.plos.org). If there are restrictions on sharing of data and/or materials, please state these. Please note that we cannot proceed with consideration of your article until this information has been declared.

Please include your updated Competing Interests statement in your cover letter; we will change the online submission form on your behalf.

Please know it is PLOS ONE policy for corresponding authors to declare, on behalf of all authors, all potential competing interests for the purposes of transparency. PLOS defines a competing interest as anything that interferes with, or could reasonably be perceived as interfering with, the full and objective presentation, peer review, editorial decision-making, or publication of research or non-research articles submitted to one of the journals. Competing interests can be financial or non-financial, professional, or personal. Competing interests can arise in relationship to an organization or another person. Please follow this link to our website for more details on competing interests: https://protect-au.mimecast.com/s/dHriC4QZgRFApQopfjbrgz?domain=journals.plos.org

**Response:** The Competing Interests statement has been update to include the additional information. It now reads:

“I have read the journal's policy and the authors of this manuscript have the following competing interests: DH is supported by a Suicide Prevention Australia National Suicide Prevention Research fellowship. MU is supported by a Discovery Early Career Researcher Award from the Australian Research Council (DE180100632). DWE declares that he receives funding from Roche Pharmaceutical for consulting on the use of the Childhood Routines Inventory-Revised (CRI-R). This does not alter our adherence to PLOS ONE policies on sharing data and materials.”

5.Thank you for stating the following financial disclosure:

[Research reported in this study was supported by Bucknell University Scholarly Development Grant awarded to DWE, a Suicide Prevention Australia National Suicide Prevention Research fellowship awarded to DH, and a Discovery Early Career Researcher Award from the Australian Research Council awarded to MU. The corresponding author had full access to all the data in the study and final responsibility for the decision to submit the report for publication. The content is solely the responsibility of the authors and has not been approved or endorsed by Suicide Prevention Australia, Bucknell University, or the Australian Research Council. The funders had no role in study design, data collection and analysis, decision to publish, or preparation of the manuscript. ].

We note that one or more of the authors is affiliated with the funding organization, indicating the funder may have had some role in the design, data collection, analysis or preparation of your manuscript for publication; in other words, the funder played an indirect role through the participation of the co-authors. If the funding organization did not play a role in the study design, data collection and analysis, decision to publish, or preparation of the manuscript and only provided financial support in the form of authors' salaries and/or research materials, please do the following:

1.	Review your statements relating to the author contributions, and ensure you have specifically and accurately indicated the role(s) that these authors had in your study. These amendments should be made in the online form.

**Response:** The information relating to author contributions and roles on the online form are updated and correct.

2.	Confirm in your cover letter that you agree with the following statement, and we will change the online submission form on your behalf:

“The funder provided support in the form of salaries for authors [insert relevant initials], but did not have any additional role in the study design, data collection and analysis, decision to publish, or preparation of the manuscript. The specific roles of these authors are articulated in the ‘author contributions’ section.”

**Response:** We confirm that funding organization did not play a role in the study design, data collection and analysis, decision to publish, or preparation of the manuscript and only provided financial support in the form of authors' salaries and/or research materials. We confirm that we agree with the statement in [2]. The following statement has been added to the Cover Letter:

“The funder provided support in the form of salaries for authors DH and MU and research materials for DWE, but did not have any additional role in the study design, data collection and analysis, decision to publish, or preparation of the manuscript. The specific roles of these authors are articulated in the ‘author contributions’ section.”

**Reviewers' comments:**

1. The data analyses of the paper are not well done. The authors claim that they did hierarchical regression analyses but based on the description of the analysis procedure, they actually did stepwise regression (see page 20). Stepwise regression is usually used in exploratory studies where researchers try to search for important predictors for the criterion variable. However, this paper is clearly a confirmatory study. I don’t see any justification for using the stepwise regression. I recommend the authors just use regular multiple regression analyses.

**Response:** The data analyses have been revised as suggested by the Reviewer. We now report the results of a regular multiple linear regression analysis.

2. The results section of the paper is very poorly written. The results section merely introduced the tables for the results; it did not describe the patterns of the results at all. In the next revision, please provide a summary of the results of each table and describe the results patterns in words (i.e., do not just present the results in tables).

**Response:** We have followed APA guidelines ([www.apastyle.apa.org](http://www.apastyle.apa.org)) for reporting the various analyses, and do summarize the results/findings for each of the main analyses. In line with the Reviewer’s recommendation, we have reviewed and revised Results reporting adding summary information where we felt it was appropriate to do so. We are happy to provide further information if the Review can please clearly identify what/how they would like the Results section to be changed. Examples of summaries for each of the main tables/analyses are provided below:

Table 2: “Overall, approximately 42–44% of the total sample met the ‘threshold for further inquiry’ for depression, and 33% met the threshold for follow-up for suicide risk due to presence of SI”.

Table 3: “Social communication difficulties were significantly correlated with all variables, with effect sizes ranging from small to medium (rp = .216–.365). Study variables were all significantly correlated with depression and SI in the expected directions, with effect sizes in the small to large range (rp = -.117–.590). Effect sizes for social communication difficulties were in the medium range for depression and SI and, as expected, SI was strongly correlated with depression. In terms of the other variables, insistence on sameness, rumination (both positively) and attentional control (negatively) were most strongly associated with depression, and attentional control (negatively) was most strongly associated with SI. Thus, all of the hypothesized constructs were found to be significantly associated with SI thereby warranting their inclusion in the linear regression analysis. ”

Table 4: “Table 4 presents the results of the linear regression model predicting SI. All hypothesized constructs were included in the model. Age was controlled for by including it in the model. The full model accounted for 43.3% of variance in SI scores, F(7, 1843) = 201.19, p < .001. In addition to depression (t = 20.81), social communication difficulties (t = 9.99), insistence on sameness (t = 6.95), attentional (t = -3.44) and inhibitory (t = 3.99) control were all identified as significant predictors of SI; however, rumination (t = -1.70) was not found to be a significant predictor of SI when entered in the model with the other variables.”

Table 5: “Thus, participants who reported some SI reported significantly greater social communication difficulties, higher levels of insistence on sameness, and lower levels of attentional and inhibitory control, than participants who did not report any SI.”

3. The authors included a lot of tables (e.g., Tables 1, 2 and 3) that are not essential for addressing the research goal. I recommend the authors put these tables in the supplementary materials.

**Response:** Table 1 presents sample demographics and is not presented in the results section, Table 2 summarizes clinical depression and suicidal ideation symptoms on the main symptom measure, and Table 3 presents means and correlations between the main study variables. We respectfully disagree with the Reviewer that these tables are better placed as Supplementary materials (which are only accessed through separate download), and our inclusion of them in the main body is consistent with standard APA style reporting. Furthermore, we do not agree that these data are not important to the research goal, and instead suggest that these data are important for informing the reader as to the nature of the sample, and the relationships between the main study variables. The inclusion of primary demographic and main variable data in the main body of the manuscript is also consistent with our own publications, and those of other studies published in PLoS One. We are therefore very hesitant and prefer not to remove these tables from the main manuscript.

4. On page 6, there is a sentence that says “ASD traits were found to significantly correlate with suicidal behaviour, and the relationship was mediated by burdensomeness and thwarted belonging….. ” Does it mean burdensomeness and thwarted belonging are two of the underlying mechanisms explaining the relationship between autism and suicidal thoughts? If yes, then why this is not addressed in the next section of the paper, which is about “mechanisms underpinning suicide risk and the autistic phenotype”?

**Response**: We agree with the Reviewer that the referenced study could have potentially been described in the section “mechanisms underpinning suicide risk and the autistic phenotype” as the study addresses a mechanism from ASD traits to suicidality based on Joiner’s (2005) theory. However, we pose that this study (Pelton & Cassidy, 2017) is better placed under the subheading “Autism phenotype as a risk factor for suicide”. First, the study examined the path from autistic traits through burdensomeness/belonging to suicidality, and therefore fits equally well within the section on the contribution of autistic traits to suicidality. Second, the next section, on mechanisms, builds an argument for the role of the autistic phenotype i.e., from those constructs associated with the diagnostic criteria of the disorder to suicidality directly. Neither belonginess nor burdensomeness fit this criteria. As examined by Pelton and Cassidy, the path from ASD traits to burdensomeness/belonging to suicidality is an indirect mediation pathway. The factors we have identified are those dimensional constructs that we think may directly predict suicidal risk. While a more complex model might be constructed that includes mediation or other pathways though factors such as belonginess/burdensomeness etc., this was beyond the scope of our study. Therefore, we believe that this section (i.e., mechanisms) is better focused on the specific constructs we test in the present study.

5. There is a typo on page 8 under the “Participants” section. It says “…. condiucted recruitment online using…. ” “Condiucted” is a typo.

**Response:** Thank you for pointing this out, now corrected.

6. On page 15, please provide a citation for Little’s MCAR test. Also on page 15, I am confused why the authors found out that the data are not MCAR but still used listwise deletion.

**Response:** The reference for MCAR is now provided. We note that there was an error in our reporting of MCAR – the non-significant p-value (p = .895) does indeed indicate that data are missing completely at random. This has now been corrected in text. We removed cases with missing data based on the recommendations by Tabachnick & Fidell. Given that data were missing at random, we have not altered this methodology and apologise for the error. The sentence now reads:

“Little’s MCAR test indicated that data were missing completely at random, p = .895 [70].”

7. On page 8, the sentence, “The aims of the present study were to examine (1) the contribution of two key clinical domains––social communication difficulties, insistence on sameness––usually considered core features of ASD, but also present across a range of other disorders, that have been associated with suicidal risk and behavior, thereby deconstructing the impact of specific ASD domains on suicide risk (assessed using DSM-5 suicidal ideation; SI)….”, is very confusing to read. Please simplify the sentence.

**Response:** We have significantly revised this sentence for clarity. The paragraph now reads:

“The aims of the present study were to examine (1) the contribution of the two core clinical domains of ASD—social communication difficulties, insistence on sameness—on suicide risk (assessed using DSM-5 suicidal ideation; SI) and (2) the additional contribution and interaction of two key dimensional constructs––cognitive control and rumination. We predict that each of the identified constructs will independently contribute to SI, controlling for depression.”

________________________________________
