# Supplementary material for: Domains of the autism phenotype, cognitive control, and rumination as transdiagnostic predictors of DSM-5 suicide risk
Source: PLoS One. 2021 Jan 22;16(1):e0245562. doi: 10.1371/journal.pone.0245562 (PMC7822649; doi:10.1371/journal.pone.0245562)
Supplement: Attachment — Submitted filename: Response to reviewers.docx [file pone.0245562.s005.docx]

Dear Editor,

Thank you for the opportunity to revise and re-submit our manuscript. Please find below a detailed response to all comments. We thank the reviewer for the additional suggestions and have revised the text accordingly. In particular, we have significantly elaborated on the interpretation of the regression analysis, as well as attending to the other three minor suggestions.

Response to Reviewer

1. First, on page 15, it says that "Little's MCAR test indicated that data were missing completely at random." Actually, non-significant test does NOT indicate the data are MCAR although it is a common misconception. A non-significance result only means that the covariance structure of the data across different missing data patterns are not heterogenous. Please refer to Yuan, Jamshidian and Kano (2018)'s paper about this. In the next revision, please correct this.

Response: The text has been revised.

Revised text: “No more than 1% (M = .303, SD = .19, Range = 0–1%) of data were missing for any questionnaire item overall, and Little’s MCAR test was not significant, p = .895 [70]. Thus, following Tabachnick and Fidell [71], cases with missing data on any of the questionnaires were deleted (n = 77, 3.8%).”

2. Second, please mention in your results section how to access your data (i.e., provide the "figshare" link).

Response: added.

Inserted text: “The data that support the findings of the study are openly available in “Figshare” at https://doi.org/10.26181/5e992fc659d7c [90].”

3. Third, on page 20, I am not sure what those "t" values are. Please clarify.

Author response: In response to the reviewer’s comment, t-statistics from the regression analysis have been deleted.

4. Finally, on page 20, please also elaborate more on your results. Interpret the regression coefficients, and explain what each coefficient means based on your research purposes.

Response: Results on p20 have been significantly elaborated upon.

Revised text: “Table 4 presents the results of the linear regression model predicting SI. All hypothesized constructs were included in the model. Age was controlled for by including it in the model. The full model accounted for 43.3% of variance in SI scores, F(7, 1843) = 201.19, p < .001. Social Communication Difficulties significantly predicted SI, with the b-weight revealing that for each unit increase in Social Communication Difficulties, SI increased by 0.085 units. Similarly, Insistence on Sameness was also identified as a significant predictor of SI, with the b-weight revealing that for each unit increase in Insistence on Sameness, SI increased by 0.012 units. Attentional and Inhibitory Control both significantly predicted SI, with the b-weights revealing that for each unit increase in Attentional Control, SI decreased by 0.015 units, and for each unit increase in Inhibitory Control, SI increased by 0.015 units. Rumination was not a significant predictor of SI when entered in the model with the other variables, with each unit increase in Rumination associated with a decrease in SI of -0.010 units. Overall, Depression made the largest contribution to SI (beta = 0.484). Comparing Social Communication to Insistence on Sameness; Social Communication Difficulties (beta = 0.192) was relatively more important than Insistence on Sameness (beta = 0.144). These two core variables shared some variance, but correlations in Table 3 reveal that these were largely independent contributions. Attentional Control (beta = -0.085) and Inhibitory Control (beta = 0.077) made similar, yet relatively smaller contributions to the model.”
