# Supplementary material for: Domains of the autism phenotype, cognitive control, and rumination as transdiagnostic predictors of DSM-5 suicide risk
Source: PLoS One. 2021 Jan 22;16(1):e0245562. doi: 10.1371/journal.pone.0245562 (PMC7822649; doi:10.1371/journal.pone.0245562)
Supplement: Attachment — Submitted filename: Response to reviewers.docx [file pone.0245562.s006.docx]

Dear Editor,

Thank you for the opportunity to revise and re-submit our manuscript. Please find below a detailed response to all comments.

Response to Reviewer

1. First, when I clicked on the link for the data (https://doi.org/10.26181/5e992fc659d7c [90].), it led me to a page with an error message. Please upload your data on the Open Science Framework (OSF) and provide the appropriate link.

Response: It is unclear why the Reviewer was unable to access these data, which were hosted on the University OP[A]L Open framework. We wonder whether the inclusion of the reference number [90] in the link caused the error message? Alternatively, it is possible that the Reviewer was unable to open the file as it was in SPSS? Nonetheless, at the Reviewer’s request we have transferred the file to OSF and have replaced the OP[A]L link with the new OSF link. We have additionally saved the file in .spss, .xlsx and .csv formats, and updated the referencing and links. We have additionally double checked and confirmed the links are working and the files are readily downloadable from OSF. The new link is: <https://doi.org/10.17605/OSF.IO/C2AP3>

2. Second, please use correct APA notations for mathematical symbols. Don't just write "beta=0.144".

Response: Replaced.
